# Current allocations and target apportionment for HIV testing and treatment services for marginalized populations: characterizing PEPFAR investment and strategy

**DOI:** 10.1002/jia2.25753

**Published:** 2021-06-30

**Authors:** Austin Jones, Brian Honermann, Elise Lankiewicz, Jennifer Sherwood, Greg Millett

**Affiliations:** ^1^ Public Policy Office amfAR the Foundation for AIDS Research Washington DC USA

**Keywords:** key populations, HIV, funding, health policy, PEPFAR, programme targets, key and vulnerable populations, policy

## Abstract

**Introduction:**

The United States President’s Emergency Plan for AIDS Relief (PEPFAR) is a large bilateral funder of the global HIV response whose policy decisions on key populations (KPs) programming determine the shape of the key populations’ response in many countries. Understanding the size and relative share of PEPFAR funds going to KPs and the connection between PEPFAR’s targets and resulting programming is crucial for successfully serving key populations.

**Methods:**

Publicly available PEPFAR budgets for key populations’ services were assessed by country and geographical region for all 52 countries with budget data in fiscal year (FY) 2020. For the 23 countries which completed a full planning process in FY 2018 and 2019, PEPFAR targets for HIV testing and treatment initiation for key populations were assessed. Expenditures for KP programming were calculated to determine whether shifts in targets translated into programming. Implementing partners were characterized by the level of specialization using the share of assigned targets made up by KPs. The average target per year and implementing partner was calculated for each KP group and indicator.

**Results:**

PEPFAR country KP budgets ranged from US$35,000 to $15.2 million, and the proportion of funding to key populations varied by region, with Eastern and Southern African countries having the lowest proportion. Between FY 2018 and 2019, the KP targets for HIV testing and treatment among KPs increased, whereas expenditures on key populations decreased from US$115.4 to $111.0 million. Of the 11 countries with an increase in HIV testing targets, seven had a decrease in KP expenditures. Of the nine countries with an increase in treatment initiation targets, five had a decrease in KP expenditures. The proportion of targets assigned to partners which do not specialize in key populations increased from FY 2018 to 2019.

**Conclusions:**

Current key population policies have not resulted in a tight connection between targets and expenditures. This includes assigning a large proportion of key populations programming to partners who do not specialize in key populations, which may weaken the performance management role of the targets. These results signal that a new approach to key populations programming is needed.

## 1 INTRODUCTION

UNAIDS estimates that 62% of new HIV infections every year are among key populations (KPs), including men who have sex with men (MSM), female sex workers (FSW), people who inject drugs (PWID), transgender people (TG), people in incarcerated settings (PIP), or their partners [1]. KP programmes – particularly in Africa – have long struggled to meet and maintain the same ARV treatment, retention and viral suppression rates, and countries with high adult HIV prevalence are experiencing a concentration of HIV infections among KPs [2, 3]. Significant barriers such as stigma, discrimination, criminalization, violence, unfriendly healthcare workers, lack of specialized services and state‐sanctioned policies undermine access to healthcare services for KPs [4, 5]. Deconstructing these barriers is critical to provide quality HIV services for all and close the inequity gap between health services delivered to KPs and those delivered to other populations.

The United States President’s Emergency Plan for AIDS Relief (PEPFAR) is the largest bilateral funder of the global HIV response, with US$5.35 billion invested across more than 50 countries in the US fiscal year (FY) 2020 [6]. In many countries, PEPFAR contributes a significant portion of the funding for HIV services for KPs [7]. While PEPFAR is not the only source of KP funding, and both domestic and external funds from donors such as the Global Fund to Fight AIDS, Tuberculosis and Malaria and others are important to track, this study focuses on the policies governing PEPFAR’s KP programming.

Like most funders and US foreign aid programmes, PEPFAR does not implement its own programmes, and instead partners with outside organizations, including non‐governmental organizations, ministries of health and multilaterals, to deliver health services. Most PEPFAR programmatic decisions – including for KPs – are made through a robust annual planning process that develops detailed budgets and targets for each country where PEPFAR provides funding [8]. In addition to being important accounting processes, these budgets and targets are intended to serve as accountability mechanisms, ensuring that PEPFAR priorities are implemented.

Despite the success of these mechanisms for PEPFAR programming in general, tracking KP programming continues to be difficult as a result of few KP‐specific indicators and limited budget and expenditure data. PEPFAR recognized the need to increase focus on KPs when it launched the Key Populations Investment Fund (KPIF) in 2016 [9]. The KPIF is a US$100 million additional investment into KP‐specific programmes meant to close some of the persistent gaps in access to services and follow the evidence that KP‐led organizations are critical partners [10, 11, 12].

This paper documents whether current PEPFAR policies ensure stated KP priorities, in the form of targets and budgets, are implemented. Recent changes in PEPFAR’s financial accounting system for budgeting and expenditures allow a more in‐depth analysis of PEPFAR’s budgets, targets and implementing organizations as they relate to KPs. We examine the scale and share of PEPFAR resources directed to KP programming, characterize the relationship between KP targets and expenditures, and assess the share of PEPFAR programming being implemented by KP‐specialized partners, defined as partners for whom KPs are a large fraction of their total targets.

## 2 METHODS

As our intent in this paper is to assess the policy decisions being made by PEPFAR in resourcing programmes for key populations (MSM, FSW, PWID, TG and PIP), we draw data directly from PEPFAR. This allows us to explore how PEPFAR approaches its programmes and prioritizes KP interventions. We assess PEPFAR budgeting decisions, expenditures and programmatic interventions – HIV testing and ARV treatment initiation targets – to characterize the organizations tasked with implementing PEPFAR programming for KPs. Note, throughout this paper, we utilize PWID as opposed to people who use drugs (PWUD) as PEPFAR programming specifically targets the injection drug use mode of transmission.

### 2.1 Data sources

PEPFAR’s annual Country/Regional Operational Plan (COP/ROP) process involves most operating units (OUs) developing detailed programme budgets and allocating funding to implementing partners. OUs are either individual country or regional programmes that are treated equivalently for PEPFAR planning purposes. For this paper, all data points of interest are available at country‐level, even when the programme is administered as a regional programme, so we refer to countries instead of OUs. Country programme budgets are combined with targets set according to PEPFAR’s Monitoring, Evaluation and Reporting (MER) system [13] that cover a range of metrics PEPFAR utilizes to monitor performance. PEPFAR separately tracks expenditures by implementing partners during the implementation phase.

#### 2.1.1 Budget data

The COP/ROP budgets are released by PEPFAR online [14]. COP budget years are implemented in the following fiscal year (FY) such that the COP2018 budget corresponds to expenditures incurred and programmes implemented in FY 2019 (October 2018 to September 2019). Throughout this paper, we will refer to the COP budgets based on their year of implementation rather than their budget year. These budget data are at the country level. PEPFAR does release implementing partner level budgets, but they are insufficiently detailed for inclusion in this analysis. Budget data were downloaded on 30 July 2020.

Following a recent change in financial information systems, budget data for FY 2020 and expenditure data since FY 2018 are categorized according to the programme’s intended beneficiary population (Females; Males; Key Populations; Orphans and Vulnerable Children; Pregnant and Breastfeeding Women; Priority Populations; and Non‐Targeted Programming), among other categorizations [15].

Budgets for 52 countries were available for FY 2020, of which 49 reported non‐zero KP budgets (Table 1).

**Table 1:** Summary of data used, for included countries

Fiscal year	Targets				Budget	Expenditures	
	HIV testing		Tx initiation				
	2018	2019	2018	2019	2020	2018	2019
Countries included	23	23	23	23	52	23	23
Countries with non‐zero KP figures	16	21	12	17	49	23	23
Completeness	83.5%	76.8%	87.1%	70.2%	–	–	–
Partners	36	69	23	68	–	–	–
Source	PEPFAR budget and target reports				PEPFAR OU budget dataset	PEPFAR Programme Expenditure Dataset	

#### 2.1.2 Target data

As part of the annual COP/ROP process, PEPFAR has historically released several reports on their plans in each country [16], including Budget and Target (B&T) Reports. B&T Reports provide country‐level MER targets as well as targets for each implementing partner in a country. Both national and partner targets were extracted for use on the PEPFAR Country/Regional Operational Plans Database developed by amfAR, and were downloaded for this analysis on 13 March 2020 [17]. Throughout this paper, except when directly assessing partner targets, we utilize the country‐level targets.

We include targets for the 23 countries who completed the full COP/ROP process in both FY 2018 and FY 2019 to enable comparisons between the years. Not all countries and regions participate in a full planning process each year, and those that do not are generally smaller programmes. B&T reports for FY 2018 and FY 2019 included disaggregated target information by age, sex and key population for many indicators. We include indicators which have country‐level targets for all included countries in both years, and are provided to both KPs and other populations. Counts of individuals receiving HIV testing services and newly initiated on ARV treatment meet these criteria. For safety reasons, PEPFAR does not release results against these disaggregated targets and thus we do not include results data [13].

Implementing mechanism agreements are not always finalized at the time PEPFAR publishes B&T reports, and mechanism targets are therefore incomplete. Country‐level targets are final, so we compare the total mechanism targets to the total country targets to assess missingness. The completeness of mechanism targets varies by indicator and year from 70.2% to 87.1%, and country‐level missingness for each indicator and year are documented in the supplement (Table S1).

Additionally, while FY2020 target data have been publicly released, PEPFAR has reduced the specificity of the targets data being released – eliminating transparency into the organizations being assigned KP‐specific targets for HIV testing and treatment initiation.

#### 2.1.3 Expenditure data

PEPFAR tracks expenditures utilizing the same financial classification system described above for budgets. These data are released at the OU level for FY2018 and FY2019. As we compare PEPFAR targets to expenditures in these years, we have limited expenditure data to the 23 countries included in the target analysis [18].

### 2.2 Analysis – funding

We calculated the proportion of FY2020 budgets allocated to any “Key Population” beneficiary class under the financial classification system. Calculations were done at the country, regional (by UN region and sub‐region [19]) and global level.

Likewise, using expenditure data, we calculated the totals and proportion of PEPFAR expenditures in FY2018 and FY2019 spent on any KP beneficiary class, and changes in expenditures over these two years by country and overall.

### 2.3 Analysis – targets

We calculated the proportion of country‐level HIV testing and treatment initiations that were targeted for KPs in both FY2018 and FY2019 as well as the percentage change between the two years by country and overall.

For each implementing mechanism, we calculate the percentage of the total targets specifically targeting KPs. Implementing mechanisms were then sorted into five bins by this percentage: <10%, 10% to 24.9%, 25% to 49.9%, 50% to 99.9% and 100%. A single mechanism was calculated to have 101% of their targets coded as KPs and was assigned to the bin with 100%. A lower percentage of targets for KPs indicates a “less‐specialized” KP implementer, whereas higher percentages are “more‐specialized” implementers. For example a partner for whom only 5% of their total HIV testing target is KPs, with the rest being other populations, would be called less‐specialized. The share of the total KP target for each indicator assigned to partners in each bin was calculated. This was done for both FY2018 and FY2019.

Finally, we calculate the average target per partner per year for each KP group for both indicators.

## 3 RESULTS

### 3.1 Key population budgets

**Figure 1:**
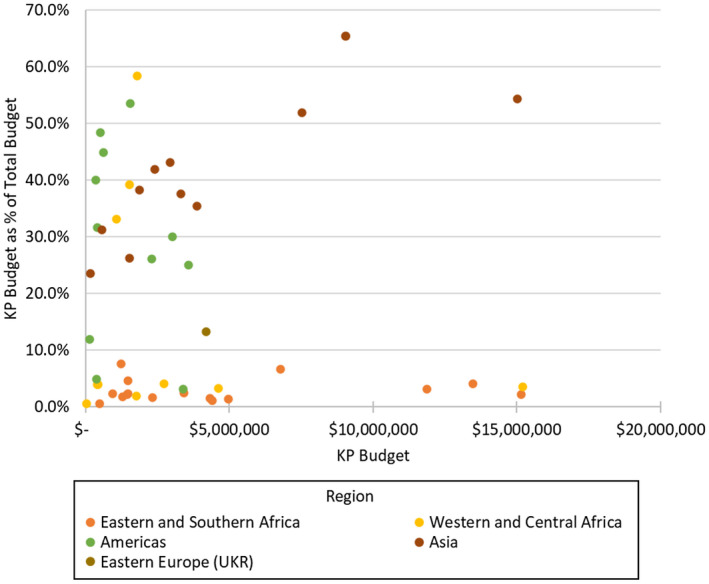
PEPFAR key populations (KP) budget as a % of total budget, fiscal year 2020.

For countries with non‐zero FY2020 budgets for KPs (n = 49), KP funding ranged from $35,000 (Ghana) to $15.2 million (Nigeria) and 0.5% (Ghana) to 65.4% (Thailand) in proportion to total COP funding (Figure 1). Three countries (Angola, Dominican Republic and Papua New Guinea) report no funds budgeted for KPs.

Countries in Eastern and Southern Africa largely had the lowest percentage of their budget allocated to KPs (2.3%) (Table 2), with Burundi (7.6%) allocating the largest share. Countries in Asia had a higher share of their budget allocated to KPs (45.6%), with Laos (23.6%) allocating the lowest share. Countries in the Americas and Western and Central Africa had countries with both high and low shares. Ukraine, the only Eastern European country, had 13.3% of its total budget allocated for KPs.

**Table 2:** Percentage of fiscal year(FY) 2020 budget allocated to key populations by geographic region

Region	FY20 KP budget (US$)	Total FY20 budget (US$)	Percent FY20 budget allocated to KP, %
Americas	16,362,686	181,482,979	9.0
Asia	45,376,321	105,857,750	42.9
Eastern and Southern Africa	78,113,909	3,231,898,174	2.4
Eastern Europe (UKR)	4,195,781	31,488,194	13.3
Western and Central Africa	29,628,107	764,286,462	3.9
Total	173,676,804	4,315,013,559	4.0

### 3.2 Key population expenditures

Reported expenditures on KPs across the 23 included countries were $115.4 million (2.96% of total PEPFAR expenditures) in FY2018 and fell to $111.0 million in FY2019 (2.85%) (Table 3).

**Table 3:** PEPFAR expenditures on key populations programming, fiscal years 2018 and 2019

Country	Key populations expenditures		
	2018	2019	% Change
Botswana	$1,454,672	$1,920,472	32.0
Burundi	$1,158,477	$1,062,428	−8.3
Cameroon	$3,414,540	$2,780,277	−18.6
Cote d'Ivoire	$4,144,377	$2,530,276	−38.9
D.R. Congo	$3,052,462	$2,723,979	−10.8
Eswatini	$1,506,402	$1,375,142	−8.7
Ethiopia	$6,952,973	$5,109,964	−26.5
Haiti	$4,194,765	$3,772,563	−10.1
Kenya	$10,636,239	$12,981,233	22.0
Lesotho	$727,055	$990,035	36.2
Malawi	$2,354,787	$2,649,461	12.5
Mozambique	$6,086,517	$4,667,548	−23.3
Namibia	$1,600,266	$1,644,721	2.8
Nigeria	$9,328,773	$6,570,685	−29.6
Rwanda	$1,303,217	$1,144,497	−12.2
South Africa	$12,122,308	$14,211,635	17.2
South Sudan	$2,231,334	$1,199,438	−46.2
Tanzania	$9,356,015	$12,132,258	29.7
Uganda	$2,516,187	$2,951,873	17.3
Ukraine	$3,859,783	$4,957,348	28.4
Vietnam	$18,162,562	$13,147,592	−27.6
Zambia	$5,913,725	$5,529,679	−6.5
Zimbabwe	$3,334,558	$4,908,176	47.2
Total (KPs)	$115,411,994	$110,961,280	−3.9
Total (all pops)	$3,900,965,380	$3,900,116,714	0.0

### 3.3 Key population targets

All 23 countries had country‐level targets for both HIV testing and treatment initiation in both years. For KPs, targets for HIV testing were found for 16 countries and across 36 implementing mechanisms in FY2018 and 21 countries and 69 mechanisms in FY2019. For treatment initiation, KP targets were found in 12 countries and across 23 mechanisms in FY2018 and 18 countries and across 68 mechanisms in FY2019.

The total KP target for HIV testing in FY2018 was 668,877 and increased to 1,268,874 in FY2019 (Table 4). In FY2018, country targets ranged from 208 in Rwanda to 225,593 in Nigeria. In FY2019, targets ranged from 757 in Lesotho to 192,693 in Kenya. KPs made up 0.87% of the total testing target (76,902,631) in FY2018 and 1.95% of the total target (65,222,881) in FY2019.

**Table 4:** PEPFAR key populations (KP) HIV testing and treatment initiation country targets, fiscal years 2018 and 2019

Country	Target: KP HIV testing services			Target: KP new on treatment		
	2018	2019	% Change	2018	2019	% Change
Botswana	2446	4296	75.6%	643	753	17.1
Burundi	12,857	18,632	44.9%	1182	1515	28.2
Cameroon	9840	11,651	18.4%	–	–	N/A
Cote d'Ivoire	10,716	38,248	256.9%	–	586	N/A
D.R. Congo	15,055	34,700	130.5%	457	1417	210.1
Eswatini	2960	2797	−5.5%	–	37	N/A
Ethiopia	34,957	–	−100.0%	17	165	870.6
Haiti	–	91,236	N/A	165	4628	2704.8
Kenya	152,662	192,693	26.2%	1409	2837	101.3
Lesotho	–	757	N/A	–	866	N/A
Malawi	4298	4060	−5.5%	475	–	−100.0
Mozambique	38,337	36,597	−4.5%	–	–	N/A
Namibia	–	1580	N/A	1177	362	−69.2
Nigeria	225,593	176,939	−21.6%	19,531	12,474	−36.1
Rwanda	208	19,548	9298.1%	–	498	N/A
South Africa	101,735	113,327	11.4%	5824	13,175	126.2
South Sudan	3095	4776	54.3%	–	2	N/A
Tanzania	–	132,624	N/A	–	17	N/A
Uganda	–	171,567	N/A	–	–	N/A
Ukraine	40,779	77,102	89.1%	–	6773	N/A
Vietnam	–	96,516	N/A	–	–	N/A
Zambia	13,339	39,228	194.1%	3698	8384	126.7
Zimbabwe	–	–	N/A	2012	3141	56.1
Total (KPs)	668,877	1,268,874	89.7%	36,590	57,630	57.5
Total (all pops)	76,902,631	65,222,881	−15.2%	3,940,535	3,262,270	−17.2

The total KP target for treatment initiation in FY2018 was 36,590 and increased to 57,630 in FY2019. In FY2018, country targets ranged from 17 in Ethiopia to 19,531 in Nigeria. In FY2019, targets ranged from 2 in South Sudan to 13,175 in South Africa. KPs made up 0.93% of the total treatment initiation target (3,940,535) in FY2018 and 1.77% of the total target (3,262,270) in FY2019.

Between FY2018 and FY2019, 11 of the 15 countries with HIV testing targets for KPs in both years had increased targets, and seven of these 11 saw a decrease in KP expenditures between the two years. For treatment initiation, nine of the eleven countries with targets for KPs in both years had increased targets in FY2019, whereas five of these nine countries saw a decrease in KP expenditures.

### 3.4 Implementing mechanism specialization

**Figure 2:**
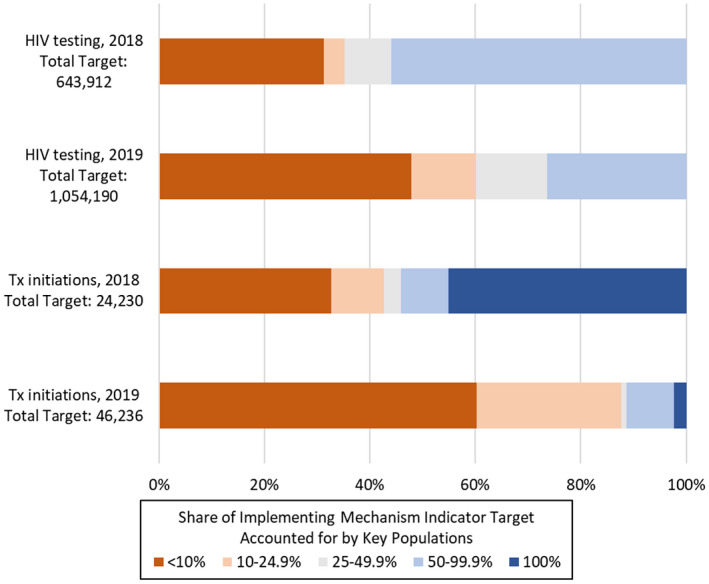
PEPFAR key populations implementer targets for HIV testing and treatment (Tx) initiation, by share of implementing partner targets accounted for by key populations, fiscal years 2018 and 2019.

In FY2018, 31.8% (201,240) of mechanism KP testing targets across included countries were assigned to the least‐specialized implementing mechanisms (n = 18), for whom KPs made up less than 10% of their total testing target, whereas 56.9% (360,180) were assigned to the most‐specialized implementing mechanisms (n = 12), for whom KPs made up more than 50% of their total testing target (Figure 2).

In FY2019, the least‐specialized mechanisms (<10% KP, n = 42) made up 49.7% (505,154) of the total KP testing target, whereas the most‐specialized mechanisms (50%+ KP, n = 14) made up 23.7% (240,365).

For treatment initiations, the least‐specialized mechanisms (<10% KP, n = 9) made up 32.7% (7928) of the total KP treatment target in FY2018, whereas the most‐specialized mechanisms (50%+ KP, n = 10) made up 54.1% (13,110). 45.1% (10,938) of the total KP treatment targets went to eight mechanisms which only had treatment targets for KPs. In FY2019, the least‐specialized mechanisms (<10% KP, n = 55) made up 59.8% (27,287) of the total KP treatment target, whereas the most‐specialized mechanisms (50%+ KP, n = 6) comprised 10.6% (5276). Purely KP treatment partners comprised only two mechanisms and 2.4% of total targets.

The average HIV testing targets per partner by KP group varied from 83.9 for TG in FY2019 to 20,713.4 for PIP in FY2018, and for treatment initiation from 5.8 for TG in FY2019 to 1457.4 for PIP in FY2019 (Table 5).

**Table 5:** Average target per implementing partner, HIV testing and treatment (Tx) Initiation, By Key Population Group (female sex workers, men who have sex with men, transgender people, people who inject drugs and people in incarcerated settings)

Population	FSW	MSM	TG	PWID	PIP
HIV testing					
2017	11,522.6	5,411.7	348.2	4,747.3	20,713.4
2018	9,125.1	2,907.5	83.9	4,393.5	7,605.8
Tx initiation					
2017	645.4	363.9	11.0	41.8	1,457.4
2018	282.3	179.0	5.8	546.1	474.1

FSW, female sex worker; MSM, men who have sex with men; TG, transgender populations; PWID, people who inject drugs; PIP, people in incarcerated settings; Tx, treatment.

## 4 DISCUSSION

The relative scale of PEPFAR’s KP investment varies dramatically by geography. Regions with higher adult HIV prevalence, especially Eastern and Southern Africa, have a smaller share of PEPFAR funds dedicated to KPs. Nevertheless, PEPFAR’s KP programmes do not match the overall epidemiology of HIV either globally or regionally. Sixty‐two percent of new HIV infections in 2019 occurred among KPs and their sexual partners, including 28% of new infections in Eastern and Southern Africa [1], a much larger share than PEPFAR’s budget and targets would indicate.

Total KP targets increased significantly from FY2018 to FY2019, whereas KP expenditures remained essentially flat. And on a country level, increases in targets more often corresponded with a decrease in expenditures. In Burundi, for example increasing KP targets for HIV testing and treatment (44.9% and 28.2% respectively) were coincident with decreasing KP expenditures (8.3%). This is consistent with previous findings that changes in PEPFAR budgets and expenditures for KPs are not closely related [20], and indicates that despite improved financial systems and an increased focus on target‐driven programming, KP targets do not seem be driving spending.

One explanation for why the targets do not seem to drive the KP programmes is that they are a small portion of the PEPFAR programme in these countries as measured by budgets and targets. If most planning and monitoring efforts are directed to the non‐KP testing and treatment programmes which make up the majority of the programme, then the attention paid to KP programmes may be insufficient to make PEPFAR’s powerful budget and target tools effective.

This same pattern is shown at the level of implementing mechanisms. In both FY2018 and FY2019, implementing mechanisms which do not specialize in KPs are responsible for a significant portion of PEPFAR’s KP HIV testing and treatment programming. Furthermore, the share of targets going to non‐specialized partners increased between FY2018 and FY2019. This increase does not necessarily represent a trend beyond these two years, but suggests a decision‐making process that can result in increased targets for KPs without increasing resources, perhaps by changing the targeted populations for a service in the planning stage and only monitoring overall results.

In the current PEPFAR system, targets are meant to drive the priorities of the implementing partners [8, 21]. This is certainly the case for some programme areas; PEPFAR does intensive performance management to push the programmes to reach targets and implements performance improvement plans for partners failing to meet aggregate targets in programming [8]. The precision and care with which these targets are set and monitored sends an implicit message about which targets are most important and which are not. In‐country PEPFAR teams receive regular updates (sometimes weekly) on the performance of indicators that drive the largest share of budgets [22]. But it is less clear whether population‐specific targets receive this attention, particularly populations that make up a small portion of a partner’s overall targets. If this accountability mechanism is not being applied to KP programming to drive performance, it is unclear which other strategies are used.

Advocates often push for both increased targets and budgets throughout the planning process [23, 24], but this analysis suggests they should be wary of increased KP targets being assigned to partners who do not focus on KPs.

Additionally, the small absolute numbers of these targets may restrict what’s possible. The treatment initiation targets for all KPs were small for several countries, and the average per‐population treatment initiation target per partner assigned such targets was even smaller. Programmes with such limited targets cannot realistically scale or develop programming and robust partnerships to do substantial outreach to these KPs, especially in the context of much larger targets and in an environment where the expectation is that partners – for good reasons – are not required to publicly document achievement against the specific KP disaggregates.

A known limiting factor for PEPFAR in monitoring KP programming like other programming is that PEPFAR’s publicly available data on KPs are limited. As noted, PEPFAR has concerns that sharing data on KPs could expose clients to security risks, and collecting that data require KPs to identify themselves as KPs – which they are not obligated to do. We share these concerns, particularly where KPs face criminalization. In the absence of robust results data, PEPFAR should develop new ways to ensure that targeted programming is actually reaching KPs.

One idea that PEPFAR has already begun to trial is using new types of partners. The PEPFAR KPIF set out to direct resources to local, KP‐led organizations [9]. This approach recognized that partners who are more deeply tied to KP communities could have a greater impact than traditional implementing partners.

Another strategy could include expanding community‐led monitoring (CLM) efforts to KP‐led organizations to do robust programmatic monitoring and quality assurance delivery specifically for KPs. CLM relies on clear, public information about what is meant to be implemented. PEPFAR should release the KP targets for both FY2020 and FY2021 as has been available in the past [16]. They should also release information on sub‐recipients, including assigned budgets and targets. CLM and parallel accountability efforts can build on these data to ensure that stated priorities like increased KP programming are in fact carried out.

There may be other implications of using non‐specialist implementing mechanisms. From the available data, we cannot say whether services are being offered in settings specifically for KPs or only in mainstream settings. Services dedicated to KPs may improve access for some individuals. Drop‐in centres for KPs, especially KP‐led, have been shown to be a preferred access point for HIV services among MSM, FSW, PWID and TG populations in several regions [10, 25, 26, 27, 28]. Others may prefer the anonymity of receiving care at facilities serving all populations, especially where doing so may allow them to avoid discrimination. Even in places where KP‐specific clinics are demanded, multiple types of access points are valuable.

This analysis is subject to a few limitations. Our definition of KP‐specialized partners is useful, but crude. It does not measure whether partners are KP‐led or trusted, nor whether they implement the programming themselves or pass these targets on to sub‐partners who may be more specialized. Information on subgrants would help here, but has not been available from PEPFAR since 2014, and even then was limited to subrecipient names [16, 21]. The link between targets for KP services and implementer spending on KPs could be tested directly with implementer‐level expenditure data. Partner expenditure data are collected by PEPFAR, but only released as aggregated country‐level figures.

The chosen measure of KP funding may exclude funds in the “Non‐Targeted Programming” classification which are used to serve KPs, but are not designated for that purpose. We measure total investment in KPs, but per‐population investment measures would be more valuable. Programmes for addressing the HIV epidemic in one KP group may be most successful if run separately from other KPs. For example MSM may be underserved if the only specialized clinics are for sex workers [27]. While the budget and expenditures datasets reported by individual KP group, the majority of KP funds in both datasets are coded “not categorized,” which limits the utility of these data. However, this may be a reflection of the programmes. Partners are limited in the number of financial categorizations (combinations of programmes, beneficiaries and cost category) they report, so the fact that many implementers aggregate rather than reporting by KP group suggests they may be responsible for multiple KP groups and non‐KP populations.

Finally, in order to study the share of targets going to KP‐specialized partners, this analysis uses only indicators that serve both KPs and other populations: HIV testing and treatment. Separate investigations into KP prevention implementation would be valuable, but require a different methodology to compare services across PEPFAR's array of prevention indicators [29].

## 5 CONCLUSIONS

PEPFAR investments in HIV services for key populations are crucial to the success of the HIV response. Turning those investments into improved services for KPs will require more than higher overall targets for KP services delivered. In the two years of implementation documented in this study, increased targets corresponded with a slight decrease in expenditures for KP programming. Instead, new funding models, partner arrangements and accountability mechanisms should be explored. The gaps in key populations’ access to HIV services will not be closed without addressing the underlying barriers that prevent services uptake in the first place. The KPIF was intended to address these structural barriers, but its implementation has changed substantially since conception [30]. Rather than directly funding KP‐led groups, the KPIF funds were passed through many of the same large implementing partners responsible for PEPFAR’s core programme, and the decision was made to evaluate KPIF programming against the same (or similar) service delivery measures as other PEPFAR programming [13, 31]. These decisions ultimately left little room and resources for KP‐led groups to focus on larger structural inequalities that undermine HIV service delivery.

## Competing interests

The authors declare no competing interests.

## Authors’ contributions

The analysis was designed by AJ and BH, with input from JS and GM. Data extraction and cleaning was conducted by BH and EL. Analysis was carried out by AJ. The first draft of the manuscript was written by AJ and revised by BH, JS and GM. All authors have read and approved the final manuscript.

## Supporting information


**Table S1**. Missingness of included indicators by country and year, calculated as the % of targets which are in the national total, but are not in the mechanism total
