# Supplementary material for: Current allocations and target apportionment for HIV testing and treatment services for marginalized populations: characterizing PEPFAR investment and strategy
Source: J Int AIDS Soc. 2021 Jun 30;24(Suppl 3):e25753. doi: 10.1002/jia2.25753 (PMC8242967; doi:10.1002/jia2.25753)
Supplement: Supplementary file 1 — Table S1. Missingness of included indicators by country and year, calculated as the % of targets which are in the national total, but are not in the mechanism total [file JIA2-24-e25753-s001.docx]

Table S1: Missingness of included indicators by country and year, calculated as the % of targets which are in the national total, but are not in the mechanism total

|  | HIV testing | | | Treatment Initiation | | |
| --- | --- | --- | --- | --- | --- | --- |
| Country | 2018 | 2019 | 2018 | | 2019 |  |
| Botswana | 0.0% | 24.5% | 0.0% | | 6.1% |  |
| Burundi | 45.4% | 4.0% | 32.7% | | 4.8% |  |
| Cameroon | 0.0% | 25.6% | 0.0% | | 31.4% |  |
| Cote d'Ivoire | 20.9% | 0.7% | 20.1% | | 0.7% |  |
| Democratic Republic of the Congo | 0.0% | 3.7% | 0.0% | | 4.5% |  |
| Eswatini | 16.3% | 15.6% | 3.5% | | 11.6% |  |
| Ethiopia | 5.5% | 9.8% | 0.0% | | 9.7% |  |
| Haiti | 18.1% | 0.0% | 12.2% | | 0.0% |  |
| Kenya | -0.5% | 8.1% | 21.5% | | 8.4% |  |
| Lesotho | 36.1% | 0.3% | 0.0% | | 0.4% |  |
| Malawi | 0.0% | 9.4% | 0.0% | | 7.9% |  |
| Mozambique | 0.1% | 5.1% | 0.0% | | 4.8% |  |
| Namibia | 0.0% | 1.6% | 0.0% | | 1.4% |  |
| Nigeria | 65.4%^1^ | 34.5% | 63.5% | | 44.6% |  |
| Rwanda | 3.2% | -0.8% | 7.4% | | -2.9% |  |
| South Africa | -0.9% | 48.9% | -0.9% | | 55.7% |  |
| South Sudan | -0.5% | 1.8% | 0.0% | | 0.0% |  |
| Tanzania | 18.0% | 40.5% | 23.4% | | 44.6% |  |
| Uganda | 30.1% | 1.3% | 36.2% | | 1.6% |  |
| Ukraine | 79.9% | 10.5% | 0.0% | | 0.0% |  |
| Vietnam | 0.0% | 0.0% | -14.6% | | -27.8% |  |
| Zambia | 27.9% | 2.0% | 24.0% | | 0.0% |  |
| Zimbabwe | -0.4% | 56.3% | -3.1% | | 53.1% |  |

^1^Nigeria does not report a total national target, so the sum of the disaggregated targets was used for this calculation.
